# Whooping Cough

**Published:** 1893-06

**Authors:** 


### WHOOPING COUGH.

This is a disease more for the mother's care than for that of the doctor. Indeed, the latter can do little. The worst part of it is, that for about two weeks you are quite unsuspicious of the fact that it has a hold upon your child, it is so like an ordinary cold. Vapo-cresoline, burned at night and during the day, if the attack is a severe one, and rubbing the chest with Roche's embrocation, are the most potent remedies. Let the diet be light, giving no meat. The severe spasms of coughing, that sometimes result in such a serious matter as rupture, may be largely ameliorated if the child is old enough to be reasoned with. Endeavor to impress upon him the necessity of self control and of expectorating the phlegm raised. The spasms will not be so severe or last so long with a child thus taught self control as with one who is of a nervous, hysterical temperament, who is allowed to give way to his feelings and become frightened with each fit of coughing.

